# Urea Derivatives as H_2_S Scavengers

**DOI:** 10.3390/molecules30040906

**Published:** 2025-02-15

**Authors:** Asger Munk Koue, Karolina Agata Szlek, Sergey Kucheryavskiy, Marco Maschietti, Christian Marcus Pedersen

**Affiliations:** 1 Department of Chemistry, University of Copenhagen, Universitetsparken 5, DK-2100 Copenhagen Ø, Denmark; 2 Section of Chemical and BioEngineering, Department of Chemistry and Bioscience, Aalborg University, Niels Bohrs Vej 8A, DK-6700 Esbjerg, Denmarksvk@bio.aau.dk (S.K.); marco@bio.aau.dk (M.M.)

**Keywords:** H_2_S scavenging, offshore industry, green chemistry

## Abstract

Simple urea-based chemicals have been used in the textile industry for “ironing-free clothes” for decades. One of the most used chemicals is 1,3-dimethylol-4,5-dihydroxyethyleneurea (DMDHEU), which consists of urea, glyoxal and formaldehyde. DMDHEU and related chemicals are considered safe and environmentally benign. We have therefore synthesized these compounds and studied their properties as H_2_S scavengers as alternatives to the “triazine” compounds used in the offshore industry today. Several derivatives are easily available, and we have evaluated their scavenging properties using Raman spectroscopy. This study reveals that this class of compounds scavenges H_2_S under conditions similar to the triazine-based scavengers and gives insight into the structural requirements needed.

## 1. Introduction

Hydrogen sulfide (H_2_S) is an extremely toxic and reactive gas with a strong, pungent smell. Therefore, H_2_S must be handled with great care and be removed when present in even tiny amounts. A common approach for removal is oxidation to elemental sulfur, which is simple and effective, but unfortunately not universally applicable [1]. H_2_S removal in the offshore oil and gas industry represents a particular challenge as the space is limited and the production flow is high under extreme conditions. The H_2_S present in the offshore production is in small amounts and originates from subsurface sulfate reduction by bacteria [2]. The H_2_S in the production stream must be removed before the oil and gas can be exported to shore. Besides its high toxicity, H_2_S readily forms metal sulfides, which can cause operational issues in production facilities, and it can be oxidized to form corroding acids. Hence, H_2_S has to be removed at various stages during production to secure a max. of 4 ppm in the oil/gas stream to prevent the formation of SO_2_ when the gas is burned and reduce toxicity [3].

**Figure 1:**
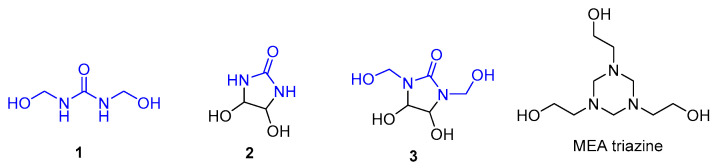
The previously studied dimethylolurea **1** (DMU) and the compounds used in the textile industry. MEA-triazine is a commonly used H_2_S scavenger.

The most common method for offshore H_2_S removal is by using scavengers, i.e., chemicals that react with H_2_S to form less harmful compounds, which ideally can be disposed of in the sea [4,5]. Therefore, water solubility is an important molecular property when developing new chemicals, which can effectively remove sulfur from the oil and gas phases to the production water, i.e., wastewater. A water-soluble compound, if not soluble in oil, can, however, not be used as a scavenger in the oil and would rely on a high pH to allow the formation of water-soluble HS^−^. In the latter case, the scavenger acts in water, and H_2_S is transferred from the oil to the water. The most frequently used H_2_S scavengers are formaldehyde derivatives. Some of them work as scavengers by releasing formaldehyde in situ, which is problematic, as formaldehyde is toxic, especially in a marine setting, making the disposal of production water to the sea unacceptable. Furthermore, formaldehyde produces solid products, which are very difficult to remove, resulting in fouling and additional operational costs [6]. Other formaldehyde-based scavengers react directly with H_2_S to form organosulfur compounds. Among these, triazines, such as MEA-triazine (1,3,5-tri-(2-hydroxyethyl)-hexahydro-*s*-triazine) (see Figure 1), are widely used due to their low cost and rapid reaction with H_2_S under basic conditions in the gas stream [7]. MEA-triazine is formulated as a basic aqueous solution and is typically administered as an aerosol. Its effectiveness relies on the deprotonation of H_2_S followed by a nucleophilic attack on the triazine core [8]. The reaction takes place in liquid droplets, making the absorption of gaseous H_2_S into the aqueous phase a key factor for a fast reaction. MEA-triazine is used in high concentrations (50–60% by mass) and in significant excess, and both unreacted scavengers and reaction products are therefore discharged with the production water. According to the OSPAR classification, triazine-based chemicals are labeled as “yellow”, indicating that they should be substituted with less harmful alternatives. In addition to the environmental concerns, triazine-based H_2_S scavengers and their reaction products are known to cause fouling and corrosion in the offshore facility, which increases costs and downtime [9].

To meet the demands for safer and more environmentally acceptable chemicals, we have developed a concept of using biomass-based compounds as scaffolds for the functional groups able to capture H_2_S. In our first approach, carbohydrate derivatives, among others, were used as scaffolds for cyanoethyl ethers [10]. The compounds were easily obtained in a few steps using water as the solvent and simple inorganic reagents as catalysts. When applied in a model study to monitor H_2_S scavenging using Raman spectroscopy [11], several of the compounds were found to be promising and, in some cases, comparable to the performance of MEA-triazine. The developed chemicals were essentially found to have very low ecotoxicity. It was, however, revealed that under scavenging conditions, acrylonitrile was released, and this was the actual scavenger, which is not in line with our efforts to develop greener scavengers.

In this article, we present our studies on using urea-based chemicals as H_2_S scavengers. We were inspired by a chemical developed by BASF in the 50s, which is used in the textile industry for maintaining the shape of clothes (iron-free) and as an experimental wood treatment [12,13]. The chemical 1,3-dimethylol-4,5-dihydroxyethyleneurea **3** (DMDHEU), is still used today and hence produced at an industrial scale and sold under the brand name “Fixapret” among others. It is synthesized from urea, glyoxal and formaldehyde and is considered to be non-toxic for humans and has very low ecotoxicity (REACH classification: EC 223-496-2). The precursors, glyoxal and formaldehyde, are toxic in a marine environment, but these are not easily formed from DMDHEU. A slow release of formaldehyde has, however, been observed when used in press-finished cotton fabrics, and the rate of release increased with pH [14]. DMDHEU is water soluble and readily biodegradable, which makes it a strong candidate for a chemical to be used offshore. In cotton fibers, it works as a cross-linker, making the textile more rigid, and we consider whether H_2_S and mercaptanes could react with DMDHEU **3** and its derivatives in a similar fashion and, thereby, remove them from the production stream. To the best of our knowledge, DMDHEU has not been studied as an H_2_S scavenger. The structurally closest compound studied is dimethylolurea **1** (DMU), a formaldehyde derivative, which has been used as a scavenger [3]. In this work, we study the use of DMDHEU and closely related compounds as H_2_S scavengers.

## 2. Results

### 2.1. Synthesis of DMDHEU and Derivatives Thereof

DMDHEU **3** is commercially available as a technical quality chemical called “Fixapret” and is used in the textile industry, but we decided to synthesize it to obtain it with high purity. The synthesis is straightforward. Glyoxal is mixed with urea to obtain the heterocyclic bis-aminal 4,5-dihydroxy-2-imidazolidinone as its trans isomer [15]. The relatively low yield mirrors the dynamic nature of the reaction, where several oligomers and, presumably also, the cis-product are formed [16]. Product **2** was then treated with formaldehyde under basic conditions to give DMDHEU **3**.

**Scheme 1:**
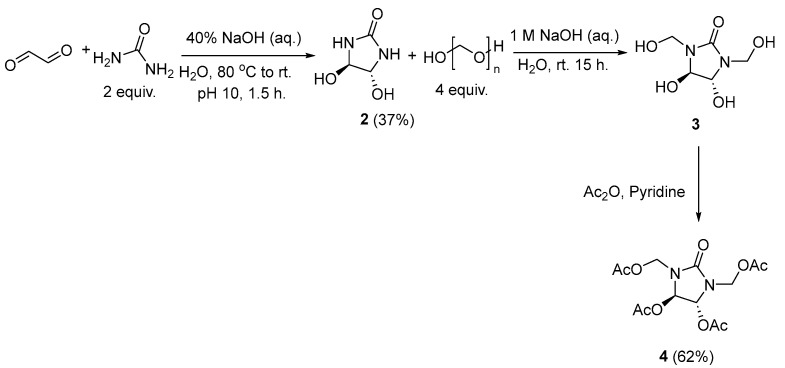
Synthesis of the intermediate (**2**) and DMDHEU (**3**). Compound **3** was acetylated to give **4**, which could be purified by chromatography and analyzed.

DMDHEU **3** is a very polar compound used as a technical product industrially. In order to obtain an analytically pure sample, a portion was acetylated to simplify purification, and spectroscopic data were obtained. These data revealed that the purity was high and that di- or oligomerization had not taken place to a significant extent. After chromatography, the acetylated DMDHEU **4** was isolated at 62%, which suggests that the crude product was of high purity and mainly contained DMDHEU and not its stereoisomers or oligomers (Scheme 1).

With access to DMDHEU, an initial screening of its reaction with H_2_S under basic conditions was carried out by mixing the scavenger with NaSH in water. The preparative experiment showed conversion of the scavenger. The product mixture, however, was found to be rather complex, and, clearly, several scavenging products were formed in the reaction. Liquid chromatography mass spectrometry (LC–MS) analysis of the crude mixture suggests that DMDHEU reacted with hydrogen sulfide up to three times, but an analytically pure sample of the main product could not be obtained (see SI for LC–MS data). The next step was, therefore, to monitor the disappearance of HS^−^ under the scavenging conditions using Raman spectroscopy, as discussed below. With this first proof of concept, we decided to make a family of compounds based on the condensation products of urea.

**Scheme 2:**
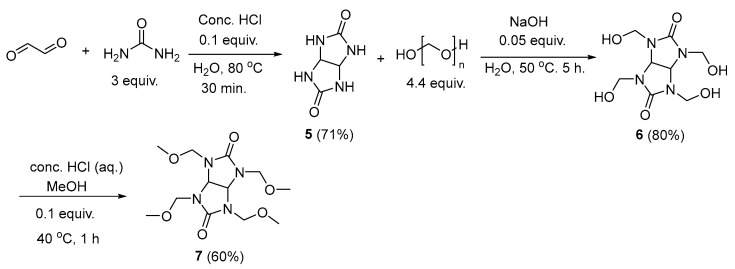
Synthesis of glycoluril (**5**) and its conversion to scavenger (**6**). Methylation of **6** to give aminal (**7**).

By changing the stoichiometry between glyoxal and urea from 1:2 to 1:3 and changing the reaction conditions to acidic, it was possible to form compound **5**, which after treatment with paraformaldehyde gave compound **6** (2,4,6,8-tetra(hydroxymethyl)glycoluril) [17]. This compound contains four hydroxyl methyl groups instead of two in DMDHEU **3**, and if these are responsible for the H_2_S scavenging, **6** should be more effective compared to DMDHEU **3**. The hydroxyl groups, additionally, increase the water solubility of the scavenger. The compound is also structurally different, being a bend bicyclic compound with cis-connected rings [18]. To study the importance of having hydroxymethyl groups in the molecule, **6** was treated with methanol under acidic conditions, giving the methoxy methylene derivative **7** in a 60% isolated yield [19]. The functional groups have now been changed from hemiaminals to hemiaminal ethers, and the water solubility properties decreased accordingly (Scheme 2).

Treating compound **6** with one equivalent of NaSH in water resulted in a complex mixture, similar to DMDHEU (**3**), but clearly a reaction was taking place, and the compounds were therefore studied in more detail using Raman spectroscopy for monitoring the kinetics of HS^−^ consumption. LC–MS analysis of the crude reaction mixtures from the scavenging reaction with **3** and **6** indicated that up to three sulfur atoms reacted with the scavenger (see Supplementary Materials). This capacity surpasses that of MEA-triazine, which under ideal conditions can react twice with HS^−^. These compounds were, therefore, studied further with Raman spectroscopy.

**Scheme 3:**
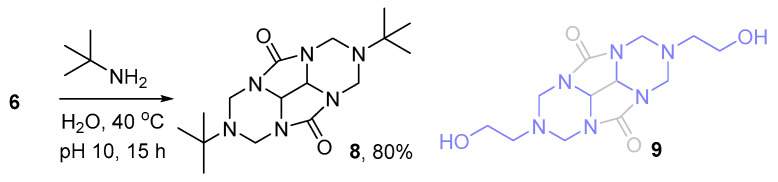
Synthesis of tetracyclic urea derivatives with two “triazine” cores. The *N*-tBu analogue **8** was synthesized in 80%, whereas the analogue **9** with hydroxyethyl groups could not be isolated. The structural motifs related to MEA-triazine are colored in blue.

The bicyclic system synthesized in Scheme 2 was extended to the tetracyclic system resembling a dimeric triazine core in MEA-triazine (Figure 1). This was achieved by reacting **6** with tert-butyl amine at pH 10 [20] (Scheme 3). The product **8** was obtained in 80% yield but was found to be insoluble in water, and the synthesis of compound **9**, resembling MEA-triazine, was therefore attempted using the same conditions, but no product was isolated, although its formation was confirmed by high-resolution mass spectrometry (HRMS).

According to the literature survey, none of the compounds synthesized above have been used for H_2_S scavenging. The structurally closest related compound was dimethylolurea (**1**, Figure 1), which is a condensation product of formaldehyde and urea. This compound is commercially available and was hence included in the study in order to have a threshold against which to evaluate the new derivatives. Dimethylolurea **1** also appears in two patents concerning H_2_S and mercaptan scavenging using combinations of chemicals [21,22].

With access to six urea derivatives, i.e., **1**, **2**, **3**, **5**, **6** and **7**, we decided to study and compare their scavenger properties in more detail. Hence, we wanted to clarify the importance of the structural elements for the scavenging properties. The reactions with NaSH were, therefore, monitored using Raman spectroscopy.

### 2.2. Raman Spectroscopic Study

A comparison of the synthesized compounds with dimethylolurea was conducted in basic aqueous solutions, which reflect the conditions used in a key industrial application where MEA-triazine solutions are injected into natural gas streams to remove H_2_S. Raman spectroscopy, which has recently been employed for the in situ monitoring of aqueous phase scavenging reactions involving bisulfide (HS^−^)—the dominant form of H_2_S at high pH—was used to monitor the progress of the scavenging reaction [11]. Bisulfide exhibits a distinct peak at 2574 cm^−1^ in the Raman spectra, which does not overlap with the peaks of MEA-triazine or its reaction products, making it suitable for monitoring the kinetics of the rate of removal of bisulfide during the progression of H_2_S scavenging. In the initial screening, it was found that the synthesized compounds, as well as their reaction products with HS^−^, were not overlapping with the signal from HS^−^, and hence, the setup could be used for this family of compounds as well.

**Scheme 4:**
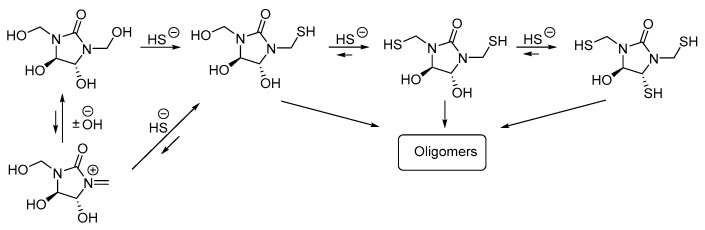
Reaction of compound **3** with bisulfide. The reaction might proceed through the iminium ion intermediate shown. Reaction products between compound **3** and up to three bisulfides have been detected using LC–MS, and oligomerization products can also be formed, making the reaction mixture rather complex.

Initially, dimethylourea **1** was tested at two temperatures, i.e., 50 and 75 °C (entries 1 and 2, Table 1). From the Raman measurements, a reduction of HS^−^ in solution could be estimated to be 20% and 50%, respectively, when applied in a 1:1 molar ratio (i.e., 100 mM bisulfide and 100 mM scavenger), hence confirming the scavenging properties of DMU reported [3]. The final pH after reaction with DMU was found to be 11.5. Compound **2** was then tested, as it could potentially react, being a hemiaminal of glyoxal. However, no decrease in HS^−^ could be detected at 25 °C, but a 30% decrease was observed at 75 °C, i.e., it is a scavenger at higher temperatures (entries 3 and 4, Table 1). This clearly suggests that the hemiaminal functionality is not very reactive under these conditions but can react with HS^−^ at higher temperatures, although less effectively compared to DMU (**1**). DMDHEU (**3**), which is the condensation product between formaldehyde and **2**, was then tested. At low temperature, the reaction with HS^−^ was found to take place with a 25% decrease, but at 75 °C, which is also more relevant to the conditions offshore, a decrease in the concentration of HS^−^ by 80% in 1 h was observed (entries 5 and 6, Table 1). Hence, DMDHEU is indeed a potent scavenger and better than DMU. Turning to the bicyclic urea derivatives, compound **5** was tested, and, as expected based on the results with compound **2**, no fast reaction with HS^−^ was observed at 50 °C (entry 7, Table 1). The formaldehyde condensation product **6**, on the other hand, reduced the concentration of HS^−^ at both temperatures (entries 8 and 9, Table 1), but to a much lower extent compared with DMDHEU **3**. This is surprising, as one would expect that a molecule containing four hydroxymethyl groups (masked formaldehydes) would outperform a molecule with only two. An advantage with compound **6** could be its ability to uptake more bisulfide for a certain scavenger volume, where fast reactions are not required. The last tested molecule, **7**, has structural similarities to the triazine-based scavengers containing methyloxymethyl residues, but in this setup, no scavenging was observed. This suggests that the scavenger is not very reactive, but the poor solubility in water does also add to the lack of scavenging, although it dissolved during the experiment. It seems, however, that the N-CH_2_OH groups are important for effective scavenging (similar to hexahydrotriazines already used in the industry) and that this functional group is significantly more reactive compared to the methylated derivatives. Based on these observations, we suggest that HS^−^ initially reacts at the hydroxymethyl, presumably via an iminium ion intermediate (Scheme 4). This reaction can then be repeated and results in products with up to three HS^−^ captured. As the reaction products also react amongst themselves, dimers and oligomers can be formed, resulting in a complex mixture of products.

## 3. Materials and Methods

### 3.1. General Synthetic Methods

All commercially available chemicals were used without further purification. Dry solvent was collected from an Innovative Technology PS-MD-05 solvent drying system (Innovative Technology, Newburyport, MA, USA) or dried with activated 4 Å molecular sieves. Thin layer chromatography (TLC) plates: silica gel (60F) coated aluminum sheets. The TLC plates were visualized by staining with either 10% solution of H_2_SO_4_ in ethanol or a Cerium Molybdate stain (Hanessian’s Stain). Reaction mixtures were concentrated in vacuo at 40 °C. Purification of products by flash column chromatography was carried out either manually by using silica gel (40–63 μm) or by using a Büchi pure C-815 flash (Flawil, Switzerland) using FlashPure EcoFlex Silica Cartridges.

Identification of compounds was performed with ^1^H-NMR and ^13^C-NMR spectra, which were recorded on a Bruker 500 MHz Ultra Shield Plus spectrograph (Billerica, MA, USA) equipped with a cryoprobe. The ^1^H-NMR was recorded at 500 MHz and the ^13^C-NMR at 126 MHz. The spectra were referenced to the chemical shifts of the deuterated solvents (CDCl_3_: ^1^H: 7.26 ppm, ^13^C: 77.16 ppm), (D_2_O: 1H: 4.79 ppm), (DMSO-*d*_6_: ^1^H: 2.500 ppm, ^13^C: 39.520 ppm). High-resolution mass spectrometry (HRMS) was performed on a Bruker SolariX XR7T ESI/MALDI-FT-ICR-MS instrument using matrix-assisted laser desorption ionization (MALDI) with dithranol as the matrix.

### 3.2. Chemical Synthesis

4,5-Dihydroxy-2-imidazolidinone (**2**) [23] was synthesized from urea (100 g, 1.67 mol), which was added to 40 wt. % glyoxal (120.80 g, 832.56 mmol) in H_2_O. The reaction mixture was heated to 80 °C until all the urea was dissolved. Then the reaction mixture was cooled to r.t., and the pH was adjusted to 10 by the addition of 40% NaOH (aq.). After stirring at r.t. for 1.5 h, a white precipitate was formed, which was filtered and washed with EtOH. Further purification was not necessary. Compound **2** (36.22 g, 306.71 mmol, 37%) was obtained as a white powder. ^1^H NMR (500 MHz, DMSO-*d*_6_) δ 7.06 (s, 2H, NH), 5.82 (s, 2H, OH), 4.59 (s, 2H, CH). ^13^C NMR (126 MHz, DMSO-*d*_6_) δ 160.3 (C=O), 83.9 (CH). HRMS (ESP+): calculated for C_3_H_6_O_3_N_2_H^+^ 119.04512 *m*/*z* found 119.04564 *m*/*z*. Spectral data were in accordance with the literature.

4,5-Dihydroxy-1,3-bis(hydroxymethyl)-2-imidazolidinone (**3**) was synthesized from compound **2** (1.00 g, 8.47 mmol) by dissolving it in 1 M NaOH (2.70 mL) with paraformaldehyde (0.56 g, 33.87 mmol). The reaction mixture was stirred at r.t. for 15 h, whereupon the reaction mixture became concentrated. No further purification was carried out to give **13** (1.50 g, 8.47 mmol, quantitative yield) as a clear syrup. ^1^H NMR (500 MHz, D_2_O) δ 5.04 (s, 2H, CH), 4.92 (d, *J* = 11.6 Hz, 2H, CH_2_), 4.73 (d, *J* = 11.6 Hz, 2H, CH_2_). ^13^C NMR (126 MHz, D_2_O) δ 157.8 (C=O), 84.7 (CH), 63.8 (CH_2_). HRMS (ESP+): calculated for C_5_H_10_O_5_N_2_Na^+^ 201.04819 *m*/*z*, found 201.04820 *m*/*z*.

4,5-Bis(acetoxy)-1,3-bis(acetoxymethyl)-2-imidazolidinone (**4**) was synthesized from **3**. To a suspension of compound **3** (0.092 g, 0.516 mmol) in pyridine (0.36 mL), acetic anhydride (0.458 g, 4.49 mmol) was added. The reaction was stirred at r.t. for 3 h, where the reaction mixture was diluted with EtOAc. The organic phase was washed twice with H_2_O and once with brine. The organic phase was dried over MgSO_4_ and concentrated. The product was purified with flash column chromatography (SiO_2_, 30% to 40% EtOAc in heptane) to give **4** (0.110 g, 0.318 mmol, 62%) as a light yellow solid. ^1^H NMR (500 MHz, DMSO-*d*_6_) δ 6.86 (s, 2H, CH), 6.03 (d, *J* = 11.3 Hz, 2H, CH_2_), 5.98 (d, *J* = 11.4 Hz, 2H, CH_2_), 2.79 (s, 6H, CH_3_), 2.73 (s, 6H, CH_3_). ^13^C NMR (126 MHz, DMSO-*d*_6_) δ 170.0 (C=O), 169.6 (C=O), 155.4 (C=O), 81.6 (CH), 66.1 (CH_2_), 20.6 (CH_3_), 20.5 (CH_3_). HRMS (MALDI+): calculated for C_13_H_18_O_9_N_2_Na^+^ 369.09045 *m*/*z*, found 369.09034 *m*/*z*.

Glycoluril (**5**) [20] was synthesized by treating urea (10.00 g, 166.51 mmol); urea was dissolved in H_2_O (20 mL) and added to 40 wt. % glyoxal (7.73 g, 54.28 mmol) in H_2_O together with conc. HCl (1.39 mL, 16.65 mmol). The reaction mixture was heated to 80 °C and stirred for 30 min, after which a white precipitate formed. The reaction mixture was cooled to r.t., and the product was filtered and washed with H_2_O and acetone. Further purification was not necessary to give **5** (5.40 g, 38.00 mmol, 71%) as a white powder. ^1^H NMR (500 MHz, DMSO-*d*_6_) δ 7.15 (s, 4H, NH), 5.23 (s, 2H, CH). ^13^C NMR (126 MHz, DMSO-*d*_6_) δ 161.2 (C=O), 64.5 (CH). HRMS (MALDI+): calculated for C_4_H_6_O_2_N_4_H^+^ 143.05645 *m*/*z*, found 143.05656 *m*/*z*. Spectral data were in accordance with the literature.

2,4,6,8-Tetra(hydroxymethyl)glycoluril (**6**) [24] was synthesized by condensation of **5** with formaldehyde. Compound 16 (5.00 g, 35.18 mmol) was dissolved in H_2_O (11 mL); afterwards, paraformaldehyde (4.62 g, 153.87 mmol) and NaOH (0.056 g, 1.41 mmol) were added to the reaction mixture. The reaction mixture was stirred at 50 °C for 5 h, after which white precipitate was formed. The product was filtered and washed with propan-2-ol. Further purification was not necessary to give **6** (7.40 g, 28.33 mmol, 80%) as a white powder. ^1^H NMR (500 MHz, DMSO-*d*_6_) δ 6.03 (s, 4H, OH), 5.50 (s, 2H, CH), 4.79 (d, *J* = 11.0 Hz, 4H, CH_2_), 4.70 (d, *J* = 11.0 Hz, 4H, CH_2_). ^13^C NMR (126 MHz, DMSO-*d*_6_) δ 157.0 (C=O), 65.8 (CH), 65.8 (CH_2_). HRMS (MALDI+): calculated for C_8_H_14_O_6_N_4_Na^+^ 285.08055 *m*/*z*, found 285.08094 *m*/*z*. Spectral data were in accordance with the literature.

1,3,4,6-Tetrakis(methoxymethyl)glycoluril (**7**) was synthesized from **6**. Conc. HCl (0.1 mL) was added to a suspension of compound **6** (2.02 g, 7.70 mmol) in MeOH (20 mL), and the reaction mixture was stirred at 40 °C for 1 h. The reaction was quenched by adding Et_3_N (2 mL) and cooled to r.t. The reaction mixture was diluted with EtOAc, whereupon the organic phase was washed twice with saturated H_2_O and once with brine. The organic phase was dried over MgSO_4_ and concentrated. Further purification was not necessary to give **7** (1.48 g, 4.65 mmol, 60%) as a white solid. *R_f_* (EtOAc) = 0.34. ^1^H NMR (500 MHz, CDCl_3_) δ 5.44 (d, *J* = 0.8 Hz, 2H, CH), 4.88 (dd, *J* = 10.9, 0.8 Hz, 4H, CH_2_), 4.78 (dd, *J* = 11.0, 0.8 Hz, 4H, CH_2_), 3.30 (d, *J* = 0.9 Hz, 12H, CH_3_). ^13^C NMR (126 MHz, CDCl_3_) δ 158.0 (C=O), 74.7 (CH_2_), 66.6 (CH), 56.1 (CH_2_). HRMS (MALDI+): calculated for C_12_H_22_O_6_N_4_Na^+^ 341.14315 *m*/*z*, found 341.14368 *m*/*z*.

2,6-Di-*tert*-butylhexahydro-2,3a,4a,6,7a,8a-hexaazacyclopenta[def]fluorene-4,8(1*H*,5*H*)-dione (**8**) [21]. NaOH (1 M) was added to a suspension of compound **6** (2.00 g, 14.07 mmol) and paraformaldehyde (1.73 g, 57.70 mmol) in H_2_O (5 mL) in order to adjust the pH to 10. The reaction mixture was stirred at 70 °C, and after 4 h, the temperature was lowered to 40 °C, and *tert*-butylamine (2.57 g, 35.18 mmol) was added to the reaction mixture. After 15 h, the reaction mixture was cooled to 0 °C, whereupon white precipitate was formed. Further purification was not necessary to give **8** (3.77 g, 11.21 mmol, 80%) as a white solid. *R_f_* (EtOAc) = 0.67. 1H NMR (500 MHz, DMSO-*d*6) δ 5.40 (s, 2H, CH), 4.73 (d, *J* = 12.1 Hz, 4H, CH2), 3.84 (d, *J* = 12.2 Hz, 4H, CH2), 1.05 (s, 18H, CH3). HRMS (MALDI+): calculated for C_16_H_28_O_2_N_6_H+ 337.23465 *m*/*z* found 337.23479 *m*/*z*. Spectral data were in accordance with the literature.

### 3.3. Raman Spectroscopy

In all experiments, the initial concentration of both HS^−^ and the scavenger was 100 mM. The HS^−^ solution was prepared using disodium sulfide about trihydrate (Na_2_S~3H_2_O, CAS 27610-45-3, VWR Chemicals, Leicestershire, UK, product ID 83756.230), and phenylacetic acid (CAS 103-82-2, Sigma-Aldrich, St. Louis, MO, USA, product ID P16621) was used as an internal standard. The pH was adjusted to 10 ± 0.2 using HCl. The scavenger solution was prepared by dissolving the scavenger in water and adjusting the pH to 10 ± 0.2 with NaOH or HCl. The scavenger was then injected into the vial containing HS^−^ and the internal standard. The reacting system was kept at controlled temperature, and the reaction was monitored using in situ Raman Spectroscopy (Rxn1, Kaiser Optical Systems, Ann Arbor, MI, USA). Raman spectra were collected at 30 s intervals by averaging three consecutive spectra with a 5 s excitation time. The Raman spectroscopy setup and spectroscopic data analysis procedure is described in further detail elsewhere [11,25].

## 4. Conclusions

Simple chemicals, based on urea, glyoxal and formaldehyde, which all can be obtained from biomass resources, have been synthesized, and their H_2_S-scavenging properties studied by preparative experiments and Raman spectroscopy. The best performing chemical, DMDHEU (**3**), is already used in the textile industry and has a low environmental impact, making it an interesting substitute for the currently used ecotoxic chemicals, like MEA-triazine and formaldehyde releasers. We have demonstrated that the condensation of formaldehyde is key for the scavenging properties and that it is not necessarily more efficient to have chemicals with more formaldehydes attached, as DMDHEU containing two hemiaminal groups outperformed compound **6** containing four. Based on this study, more sophisticated chemicals with tailored properties can be easily achieved, as demonstrated by the synthesis of the tetracyclic compound **8**.

## Figures and Tables

**Table 1:** Scavenging properties estimated by Raman spectroscopy, where the concentration of HS^−^ in aqueous phase is monitored over time. Initial concentration of HS^−^: 100 mM. Initial concentration of the scavengers: 100 mM. Initial pH: 10. The plots for all experiments are reported in the Supplementary Information.

Entry	Compound	Temperature/°C	Percentage Decrease in HS^−^ Concentration in 1 h
1	DMU **1**	50	20% *
2	DMU **1**	75	50%
3	**2**	25	0%
4	**2**	75	30%
5	DMDHEU **3**	25	25%
6	DMDHEU **3**	75	80%
7	**5**	50	0%
8	**6**	50	13%
9	**6**	75	20%
10	**7**	50	0%

* 30% after additional 1 h.

## Data Availability

Data are contained within the article and Supplementary Materials.

## Supplementary Materials

The following supporting information can be downloaded at https://www.mdpi.com/article/10.3390/molecules30040906/s1: Kinetic data, NMR spectra and LC-MS data.
