# Supplementary material for: Urea Derivatives as H2S Scavengers
Source: Molecules. 2025 Feb 15;30(4):906. doi: 10.3390/molecules30040906 (PMC11858773; doi:10.3390/molecules30040906)
Supplement: Supplementary file 1 [file molecules-30-00906-s001.zip › molecules-3454560-supplementary.pdf]

Urea derivatives as H_2S Scavengers

Asger Munk Koue[1], Karolina Szlek,[2] Sergey Kucheryavskiy,[2] Marco Maschietti[2] and Christian Marcus Pedersen[1,*]

Contents

Kinetics of removal of bisulfide in aqueous phase via in situ Raman Spectroscopy 2

NMR of compounds data 5

LCMS data of compound **3** 18

LCMS Data of compound **3** after reaction with HS^- 19

Kinetics of removal of bisulfide in aqueous phase via in situ Raman Spectroscopy

Figure S1 shows the trends of the concentration of bisulfide in basic aqueous solutions using the compounds **2** and **3** at 25 °C. As can be seen, no significative reduction of concentration of bisulfide was observed with **2**, while approximately 25% of the initial bisulfide was scavengers by compound **3** in 1 hour.

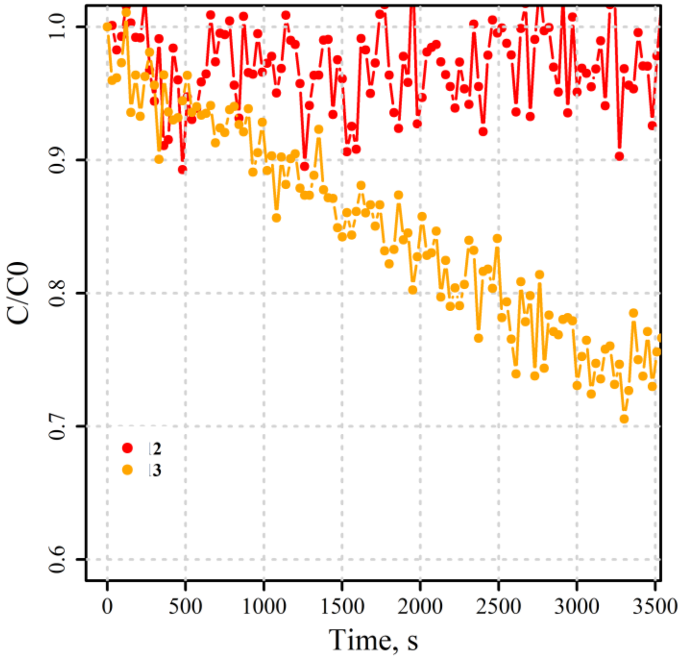

Figure S1. Trend of the concentration of bisulfide in the tests with compounds **2** and **3** at 25 °C. C is the concentration of bisulfide over time, while C0 is the initial concentration of bisulfide. Initial concentration of bisulfide: 100 mM. Initial concentration of **2** and **3**: 100 mM. Initial pH: 10. Each experiment lasted for 1 hour.

Figure S2 shows the trends of the concentration of bisulfide in basic aqueous solutions using the compounds **DMU 1** and **6** at 50 °C. The initial pH was fixed to 10. The reactions lasted for 1 hour. The pH after 1 hour was 11.5 for **DMU** and 11.0 for **6**. Compounds **5** and **7** were also tested at 50 °C; however, they did not reduce the bisulfide peak to the same degree.

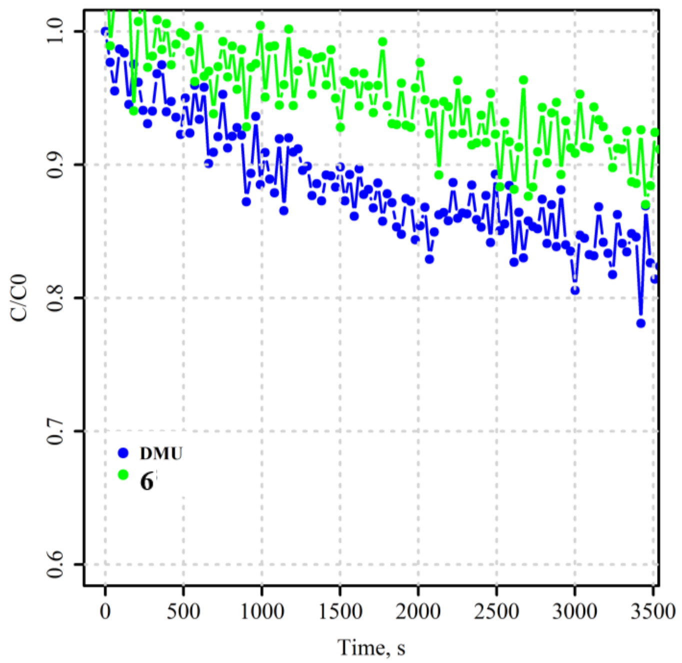

Figure S2. Trend of the concentration of bisulfide in the tests with compounds **DMU 1** and **7** at 50 °C. C is the concentration of bisulfide over time, while C0 is the initial concentration of bisulfide. Initial concentration of bisulfide: 100 mM. Initial concentration of **DMU** and **6**: 100 mM. Initial pH: 10. Each experiment lasted for 1 hour.

Figure S3 shows the trends of the concentration of bisulfide in basic aqueous solutions using the compounds **DMU 1, 2, 3 and 6** at 75 °C. The initial pH was fixed to 10. The reactions lasted for 1 hour. The pH after 1 hour was: 11.5 for **DMU 1**; 9.3 for **2**; 11.1 for **3**; and 11.5 for **6**.

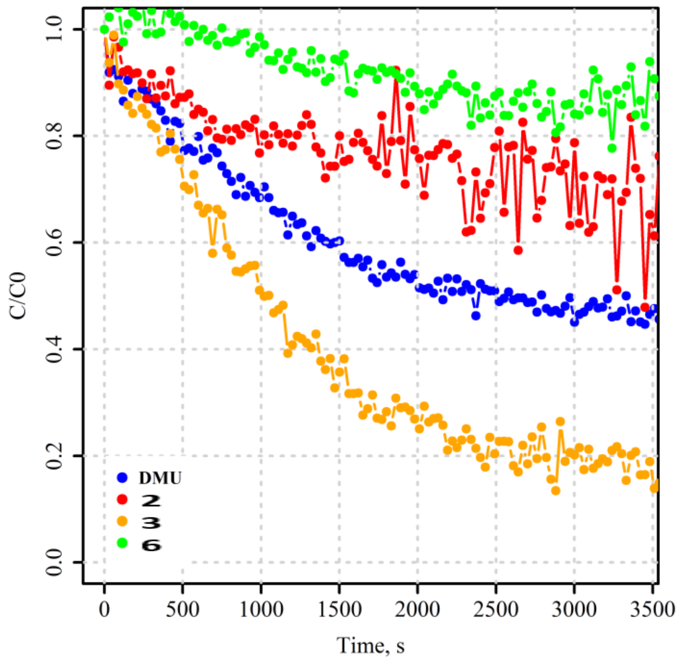

Figure S3. Trend of the concentration of bisulfide in the tests with compounds **DMU 1**, **2**, **3**, and **6** at 75 °C. C is the concentration of bisulfide over time, while C0 is the initial concentration of bisulfide. Initial concentration of bisulfide: 100 mM. Initial concentration of **DMU 1**, **2**, **3**, and **6**: 100 mM. Initial pH: 10. Each experiment lasted for 1 hour.

NMR of compounds data

4,5-Dihydroxy-2-imidazolidinone (2)

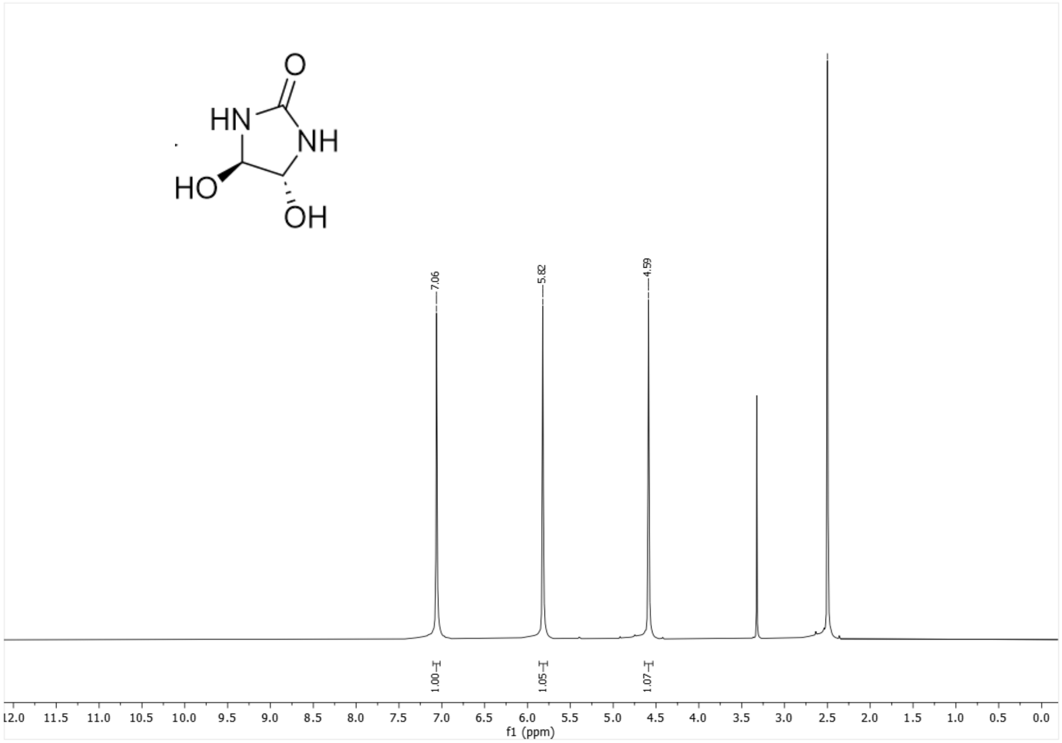

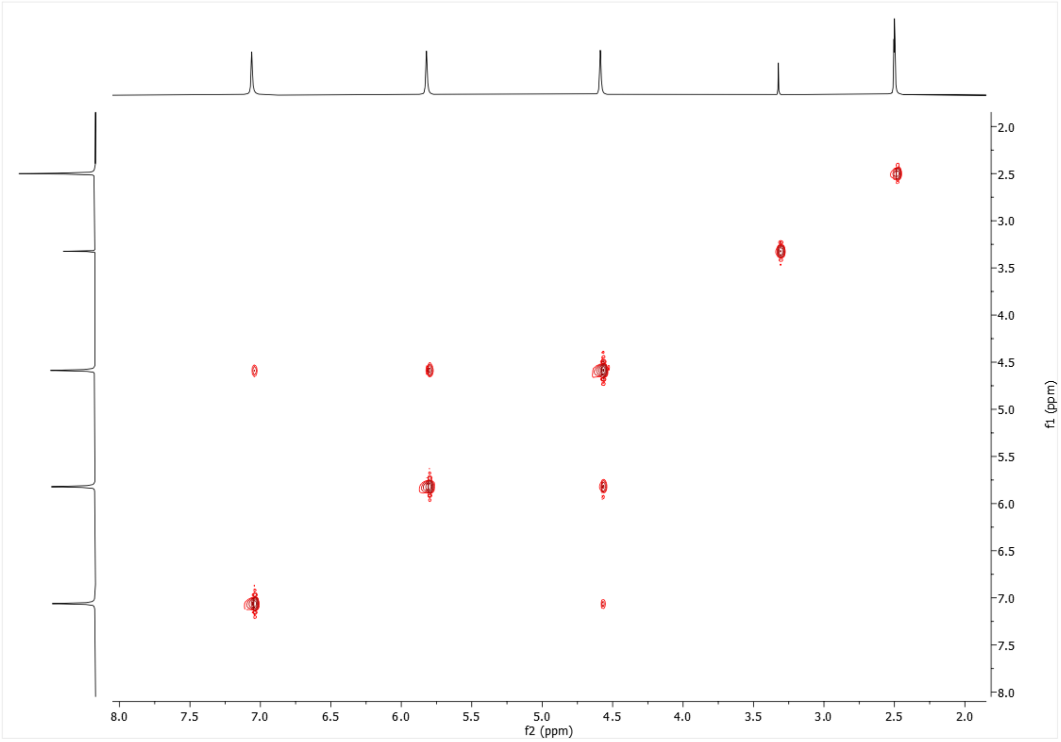

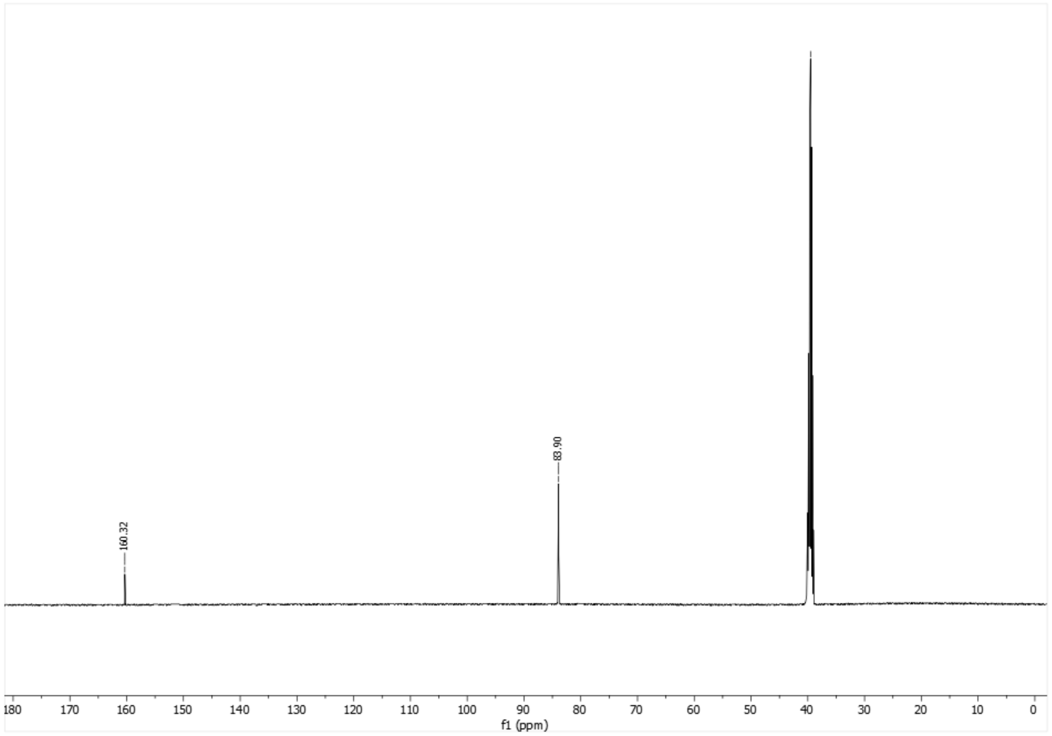
160.32
83.90
180
170
160
150
140
130
120
110
100
90
80
70
60
50
40
30
20
10
0
f1 (ppm)

4,5-Dihydroxy-1,3-bis(hydroxymethyl)-2-imidazolidinone (3)

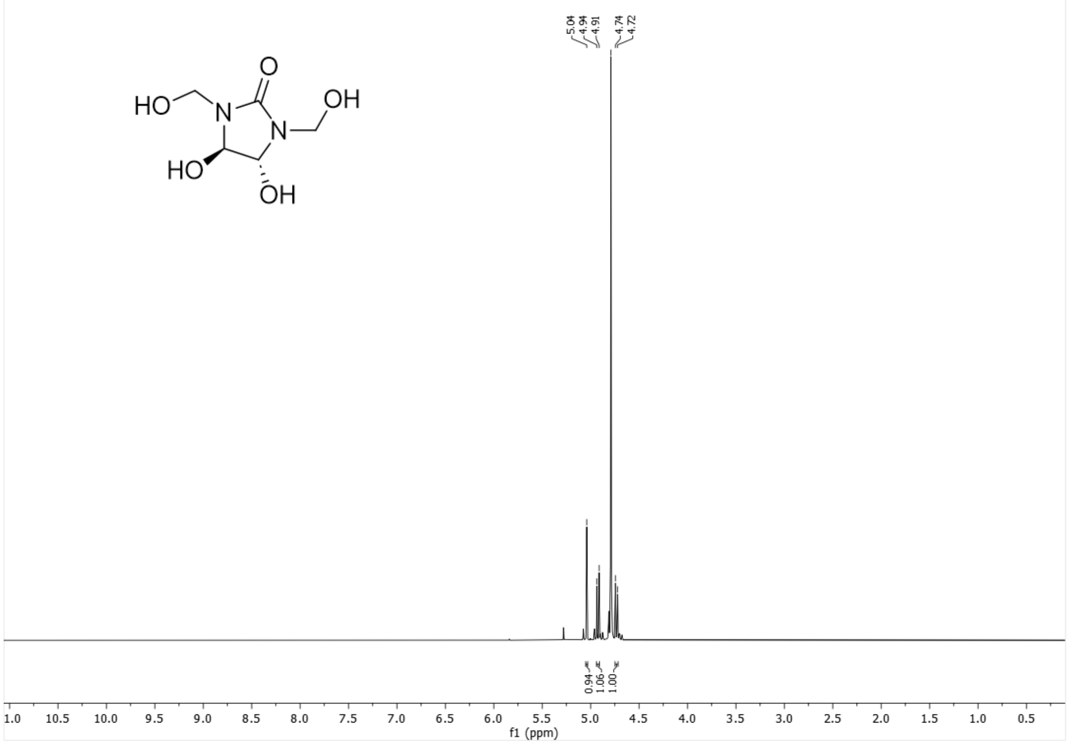

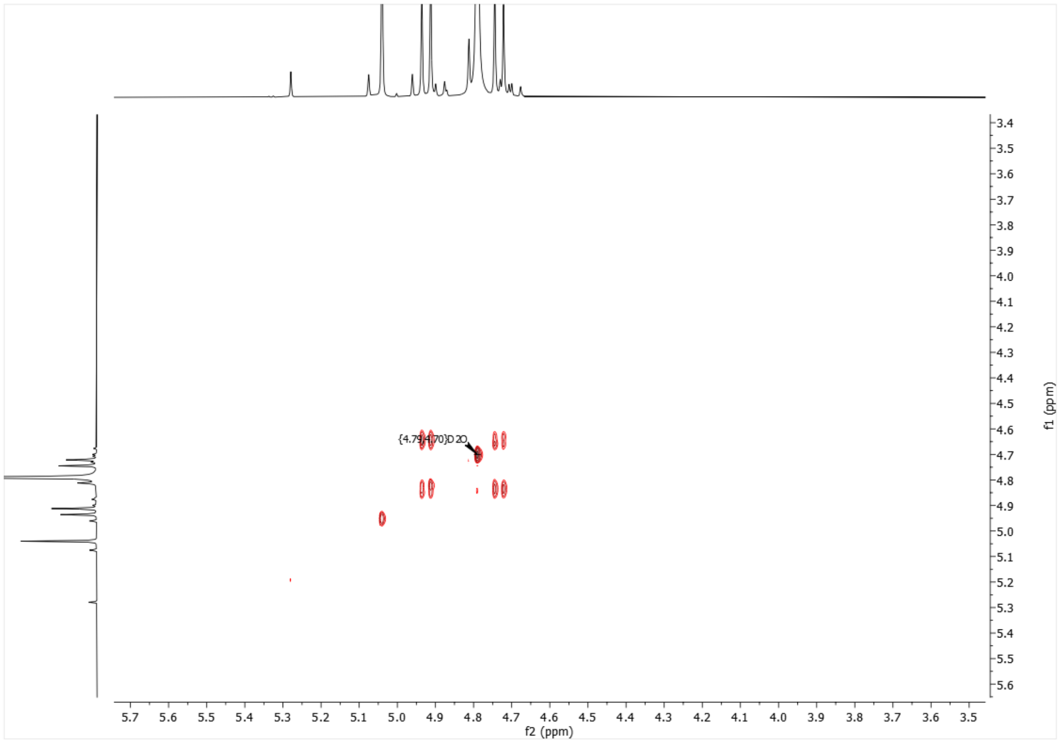

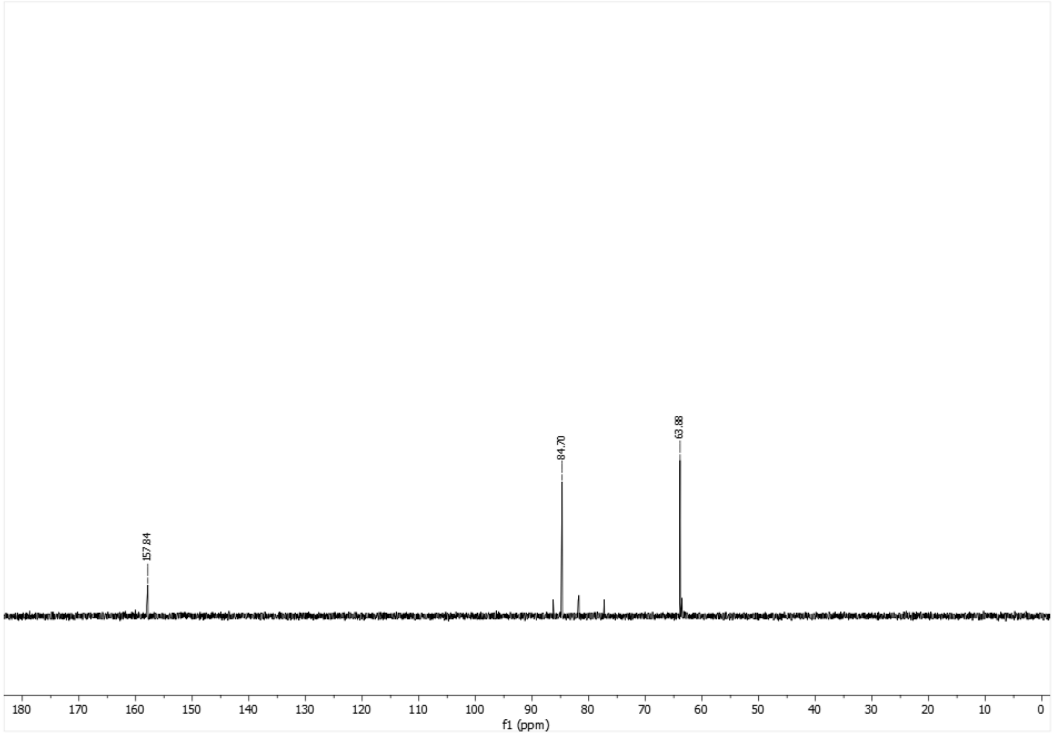
157.84
84.70
63.88
180
170
160
150
140
130
120
110
100
90
80
70
60
50
40
30
20
10
0
f1 (ppm)

4,5-Bis(acetoxy)-1,3-bis(acetoxymethyl)-2-imidazolidinone (4)

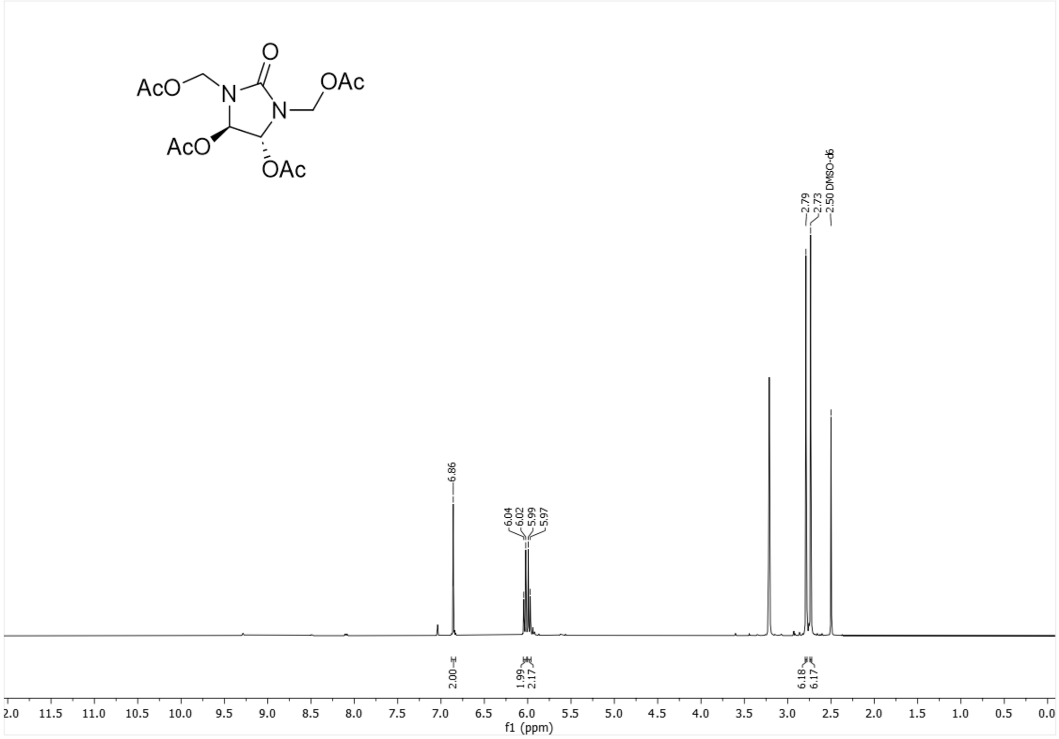

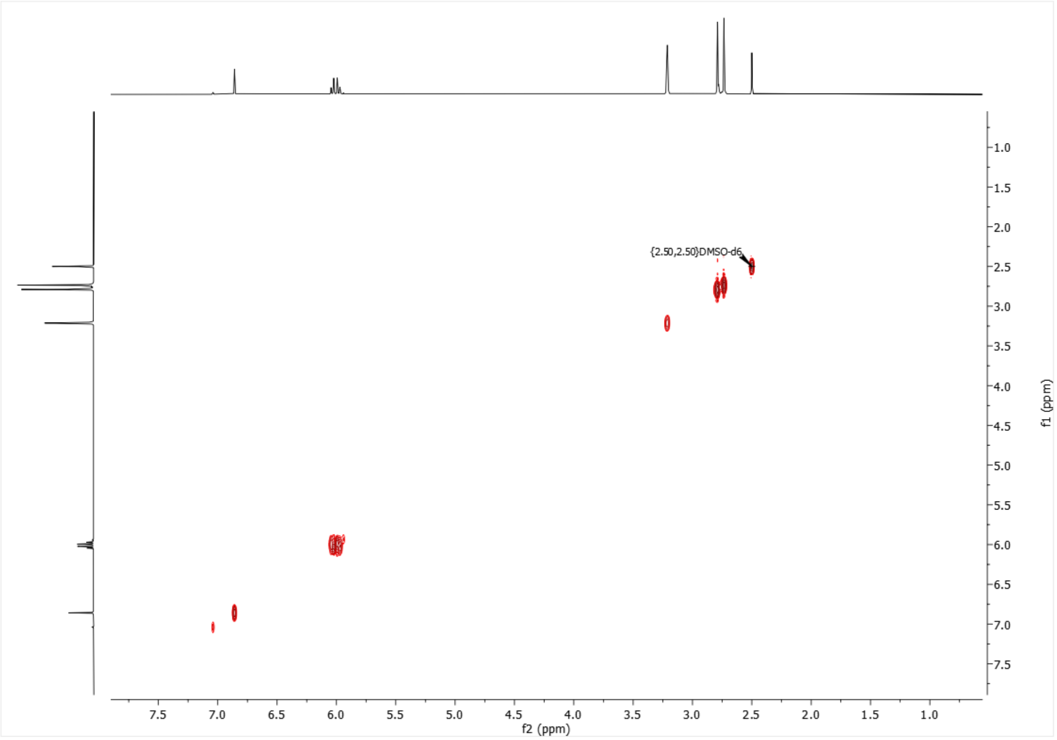

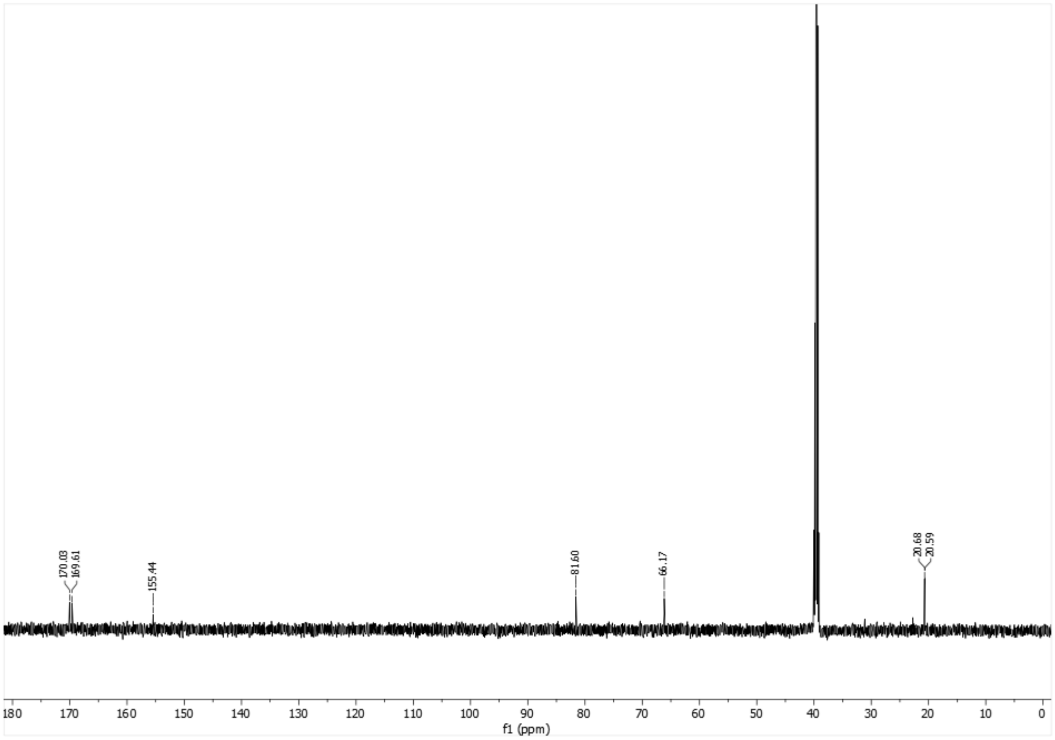
170.03
169.61
155.44
81.60
66.17
20.68
20.59
180
170
160
150
140
130
120
110
100
90
80
70
60
50
40
30
20
10
0
f1 (ppm)

Glycoluril (5)

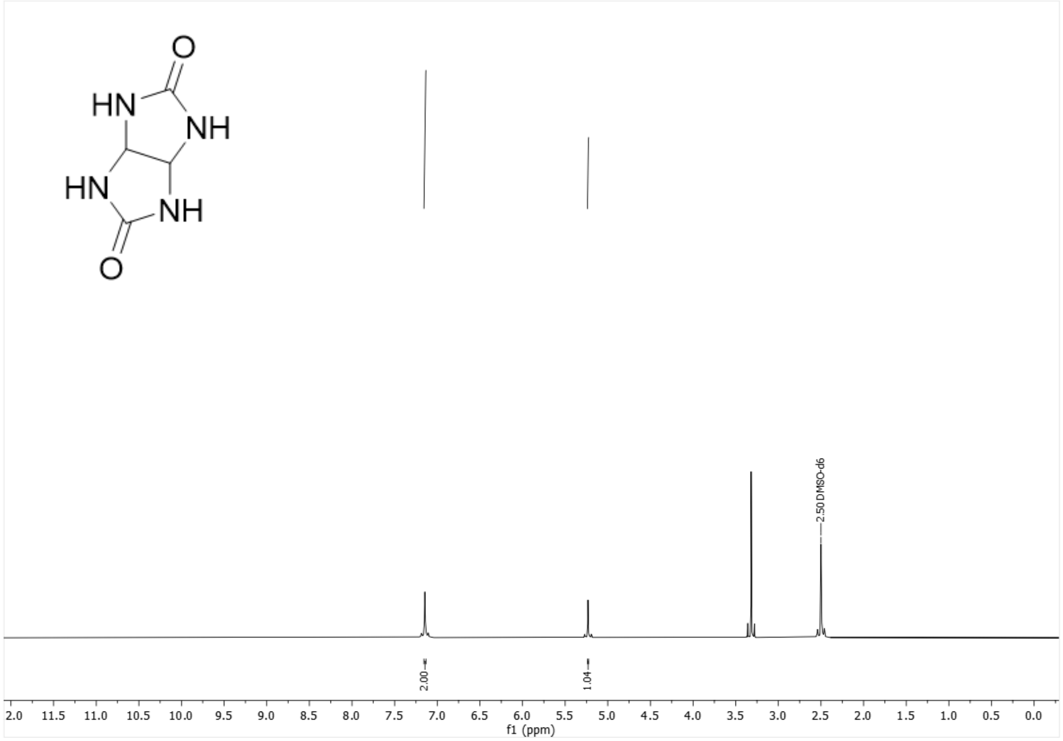

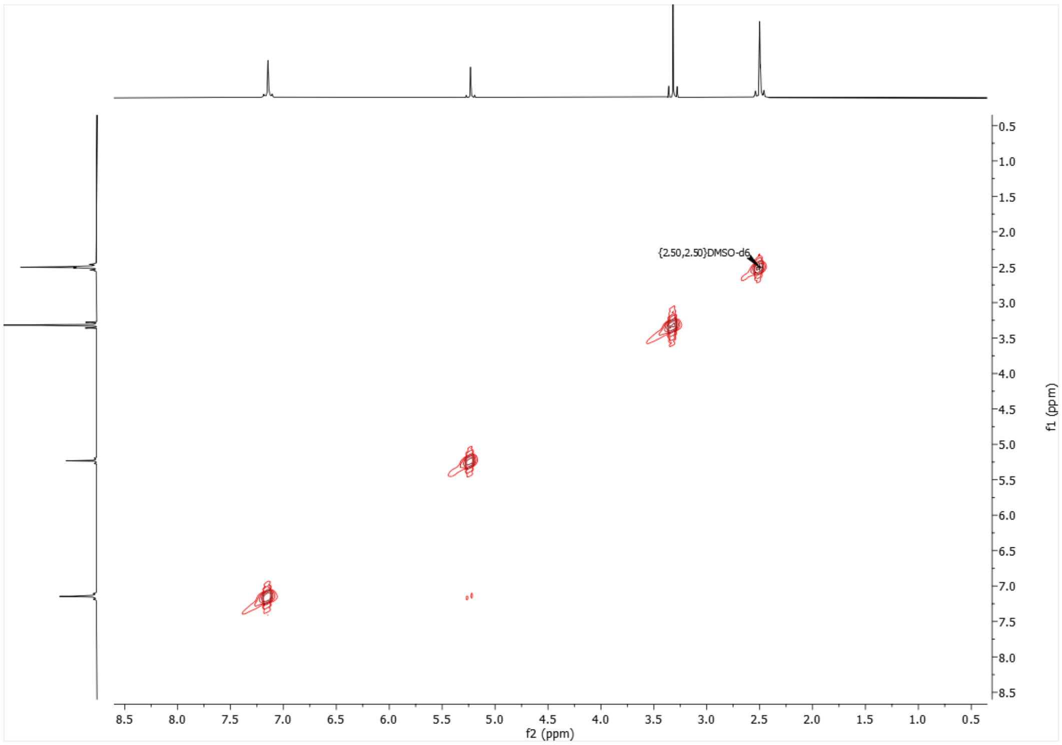

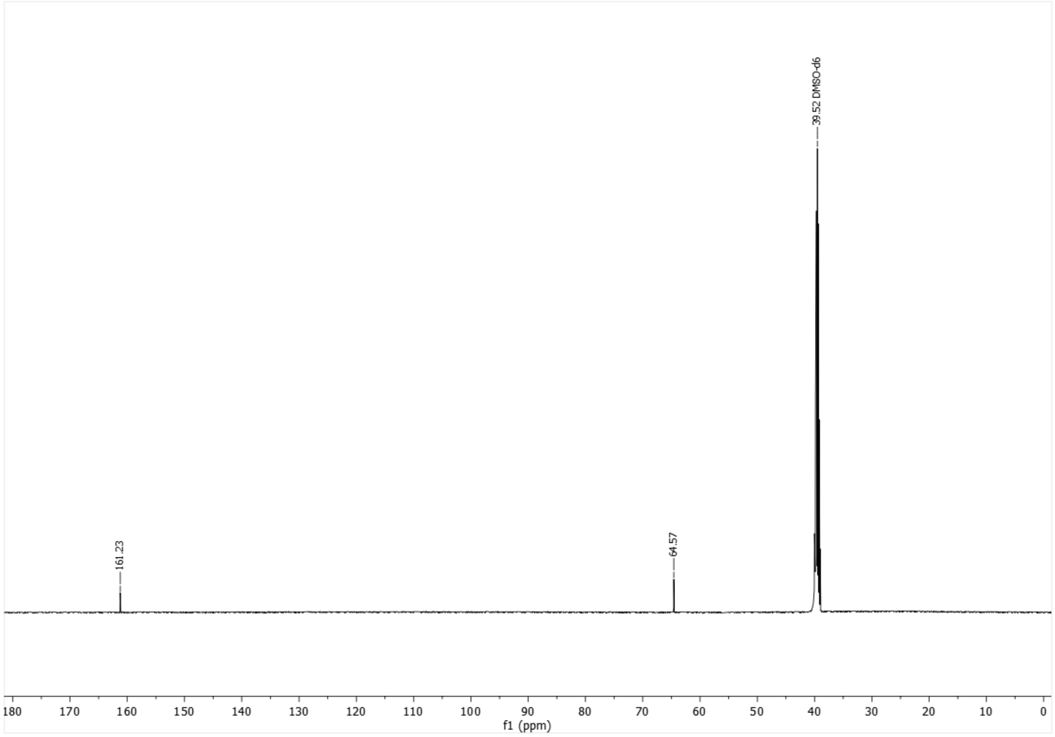

39.52 DMSO-d6
161.23
64.57
180
170
160
150
140
130
120
110
100
90
80
70
60
50
40
30
20
10
0
f1 (ppm)

2,4,6,8-Tetra(hydroxymethyl)glycoluril (6)

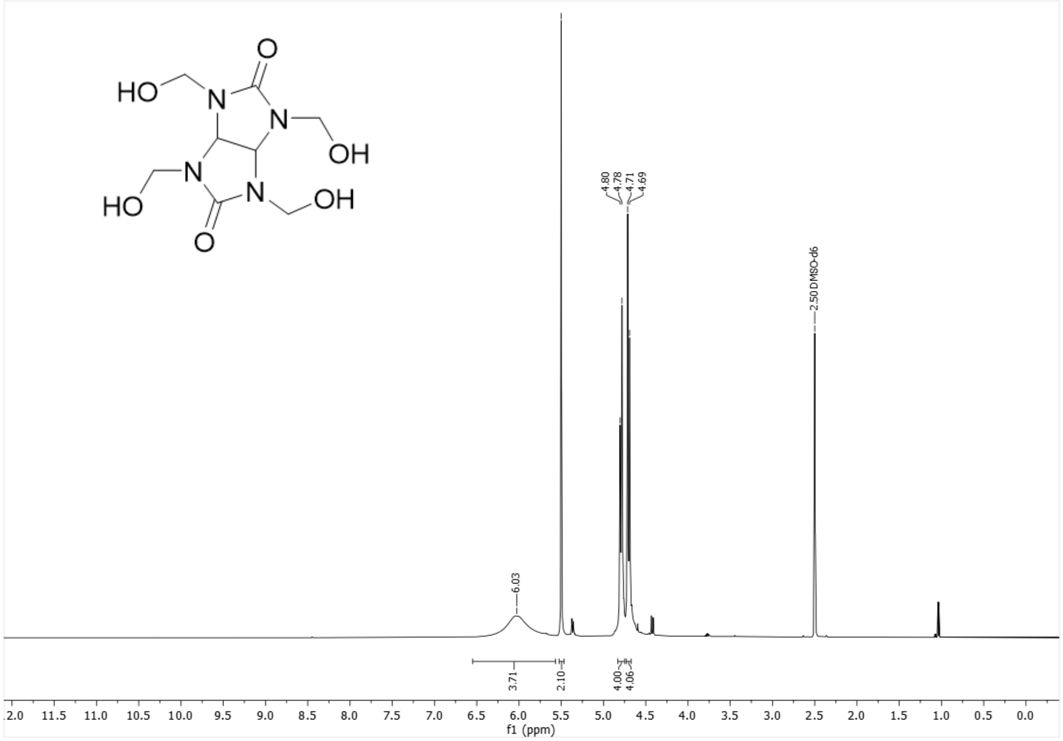

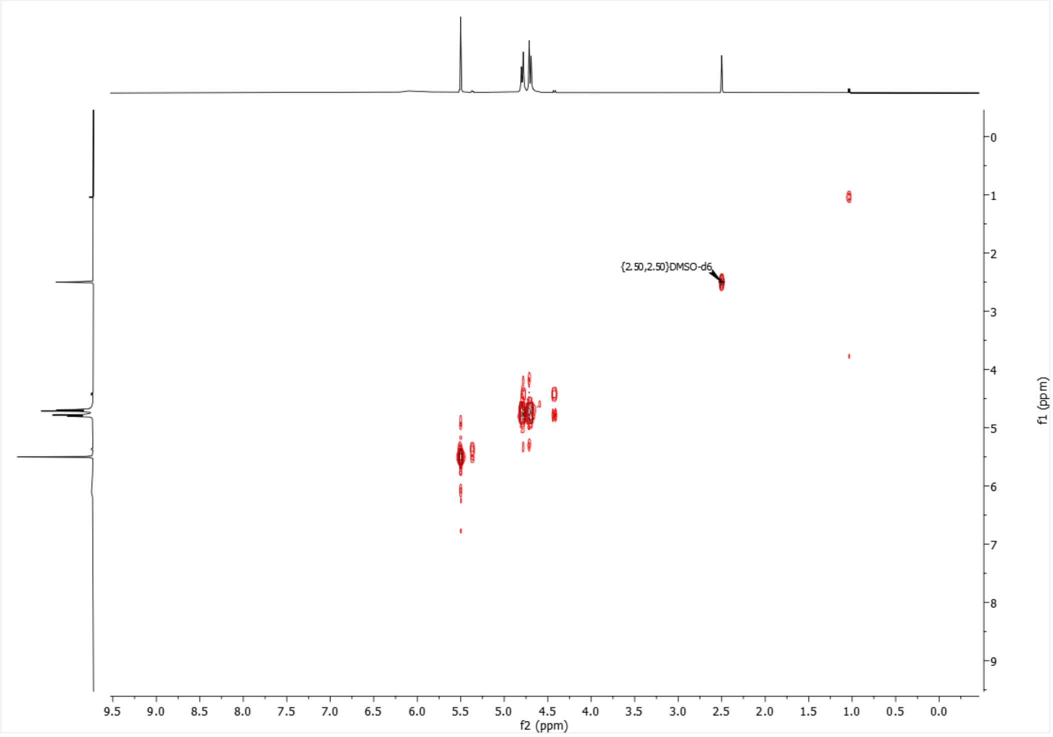

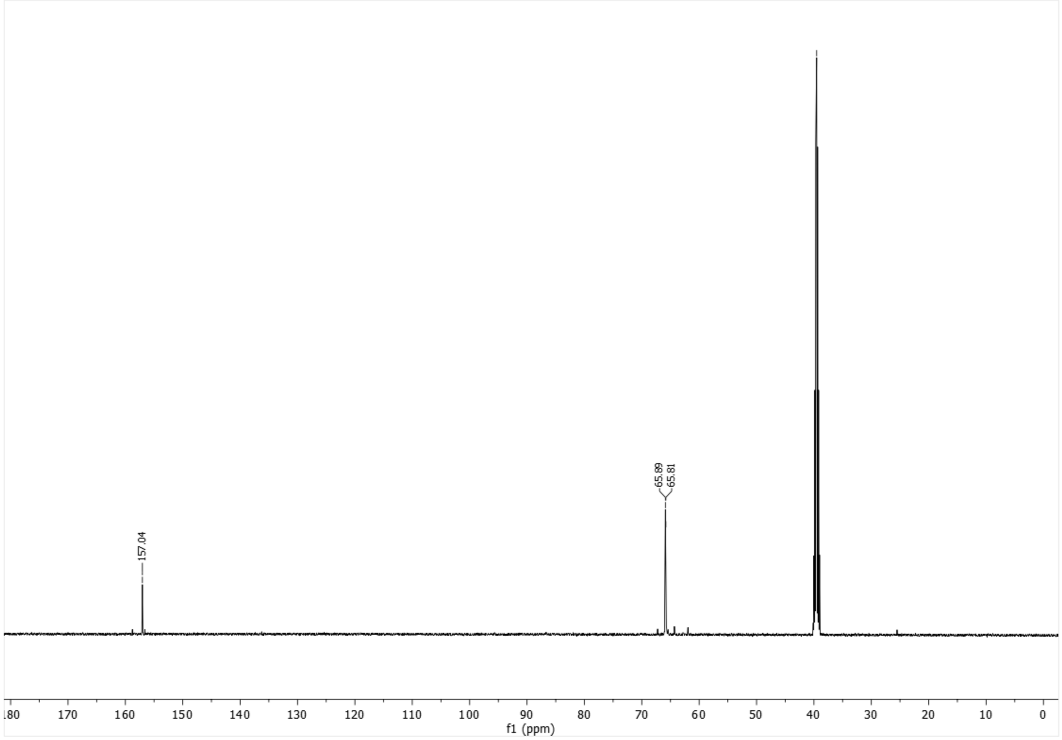
157.04
65.89
65.81
180
170
160
150
140
130
120
110
100
90
80
70
60
50
40
30
20
10
0
f1 (ppm)

1,3,4,6-Tetrakis(methoxymethyl)glycoluril (7)

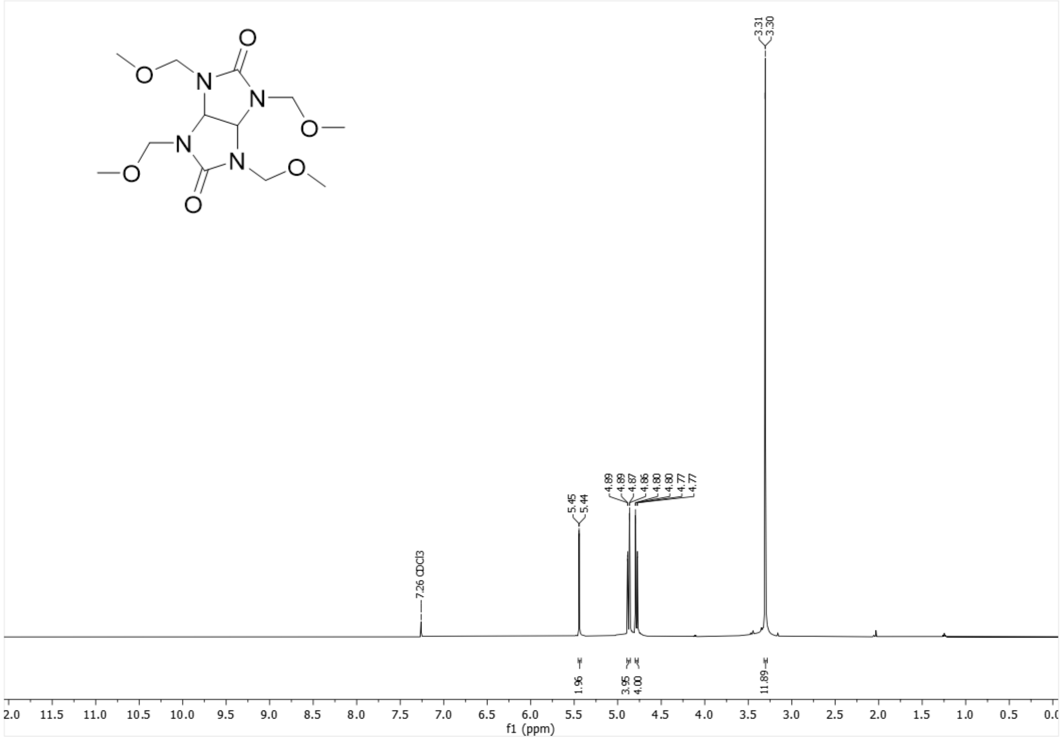

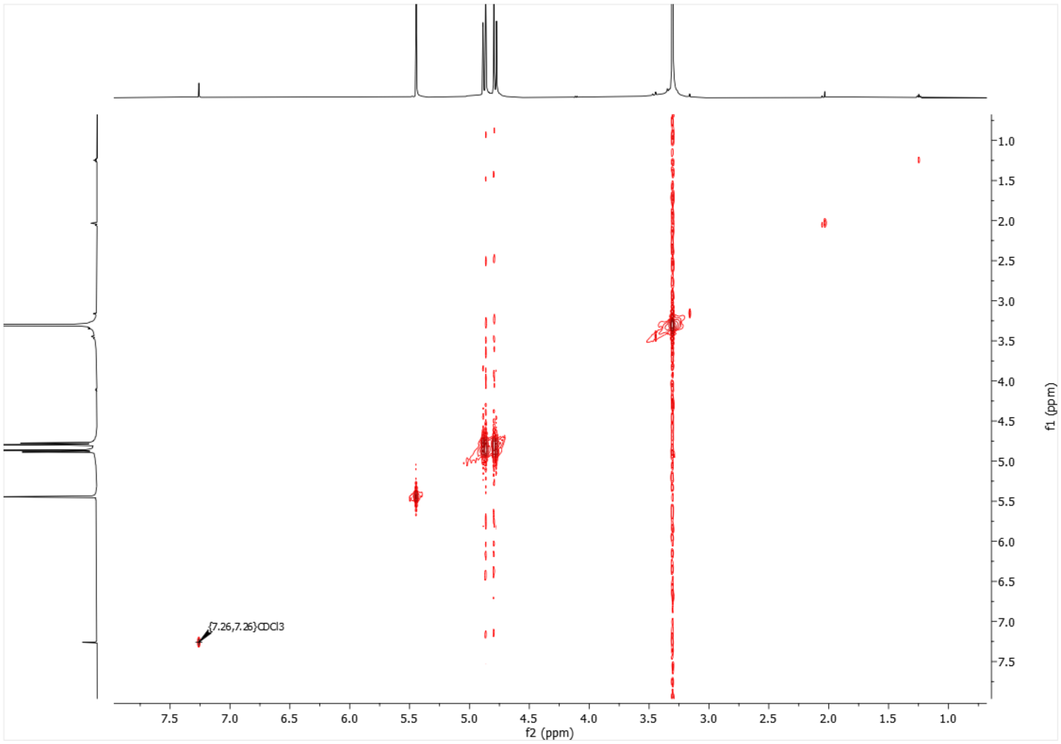

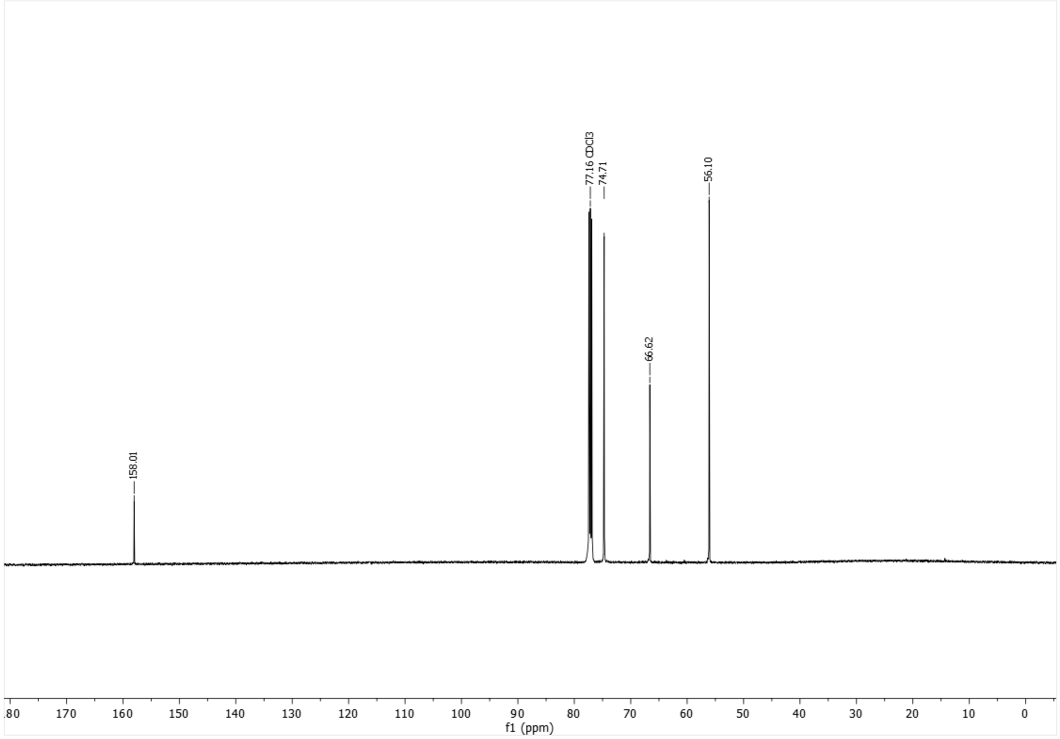
77.16 CDCl3
74.71
56.10
66.62
158.01
f1 (ppm)

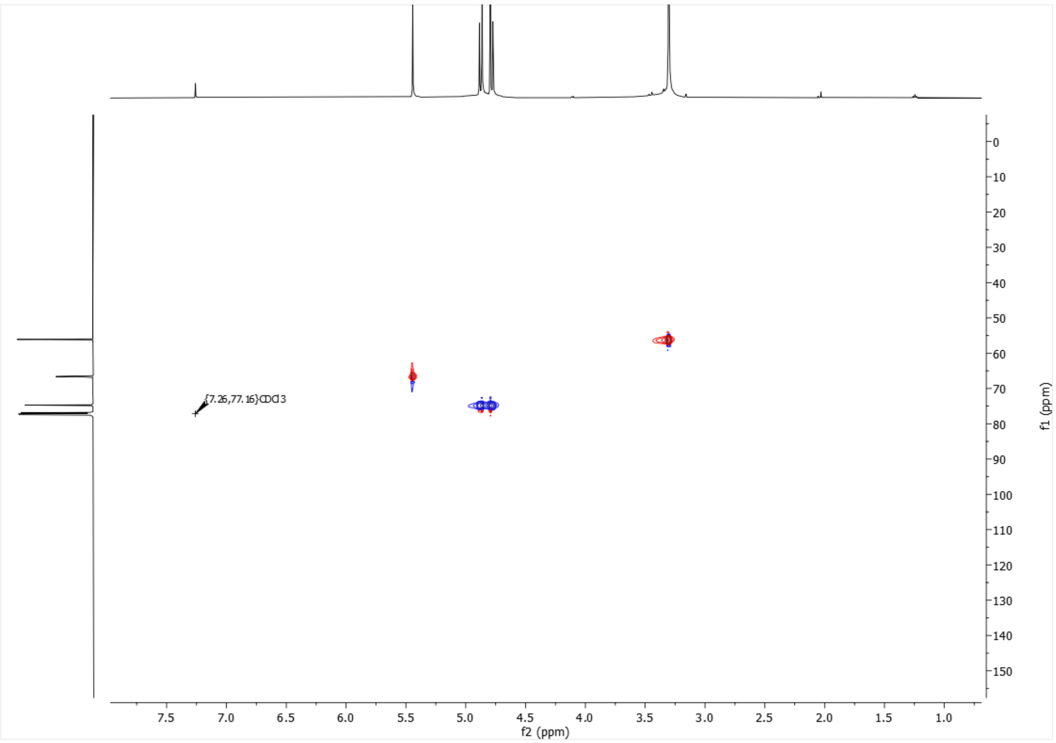
{7.26,77.16}CDCl3
f2 (ppm)
f1 (ppm)

2,6-Di-tert-butylhexahydro-2,3a,4a,6,7a,8a-hexaazacyclopenta[def]fluorene-4,8(1*H*,5*H*)-dione (8)

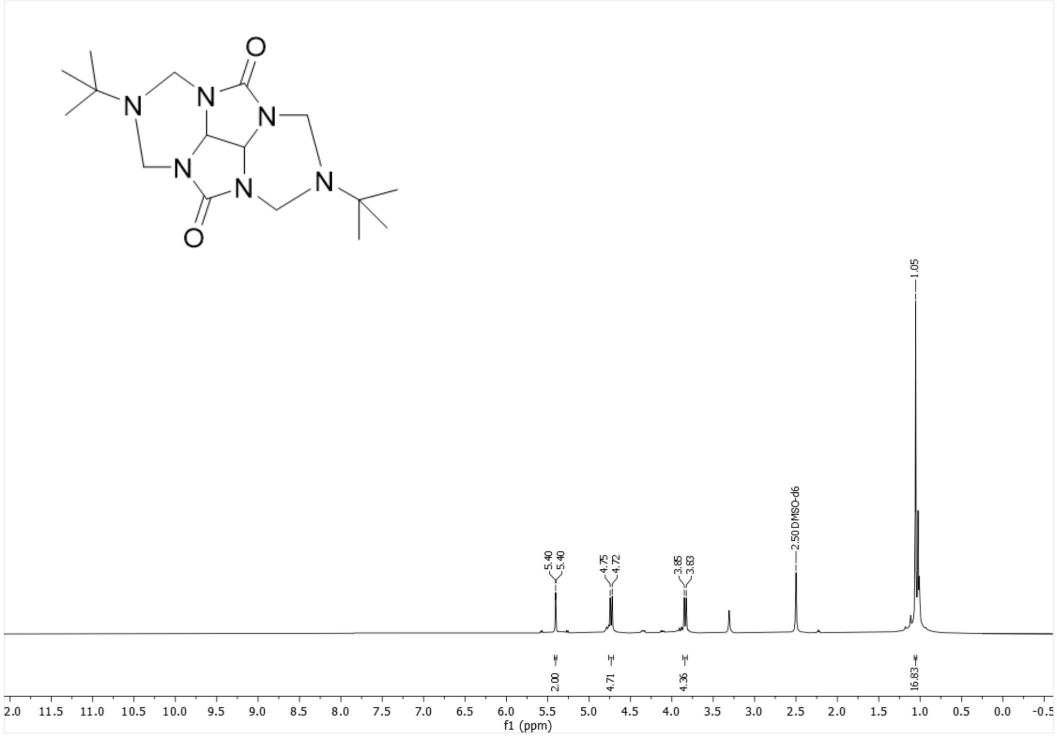

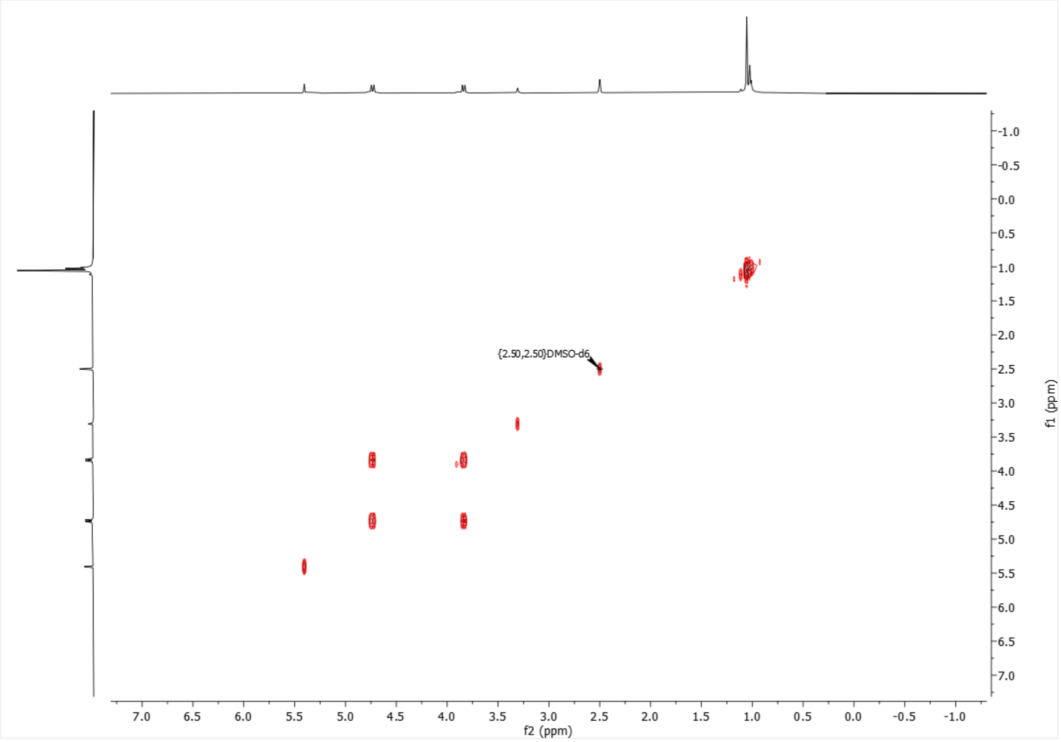

LCMS data of compound **3**

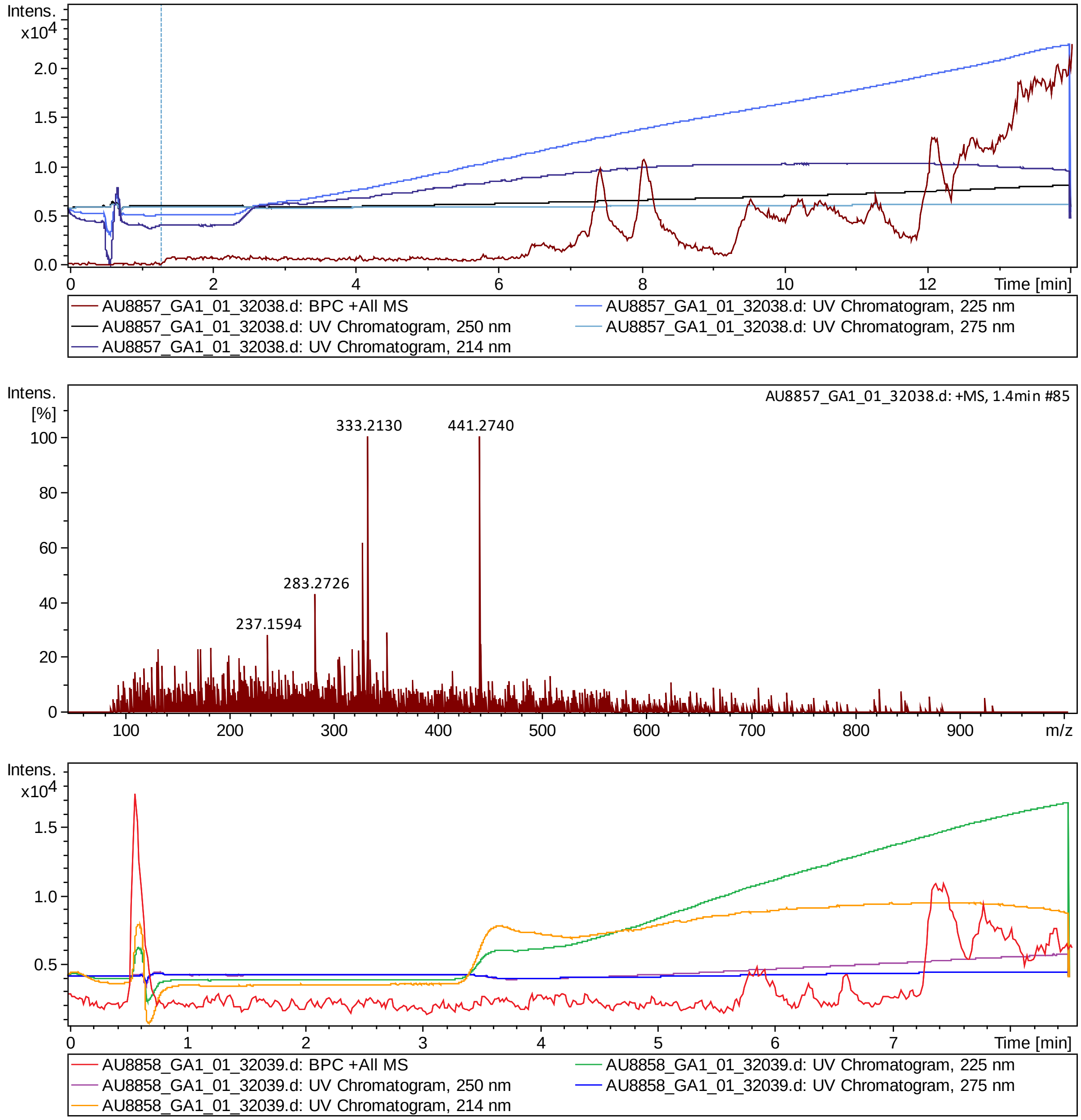

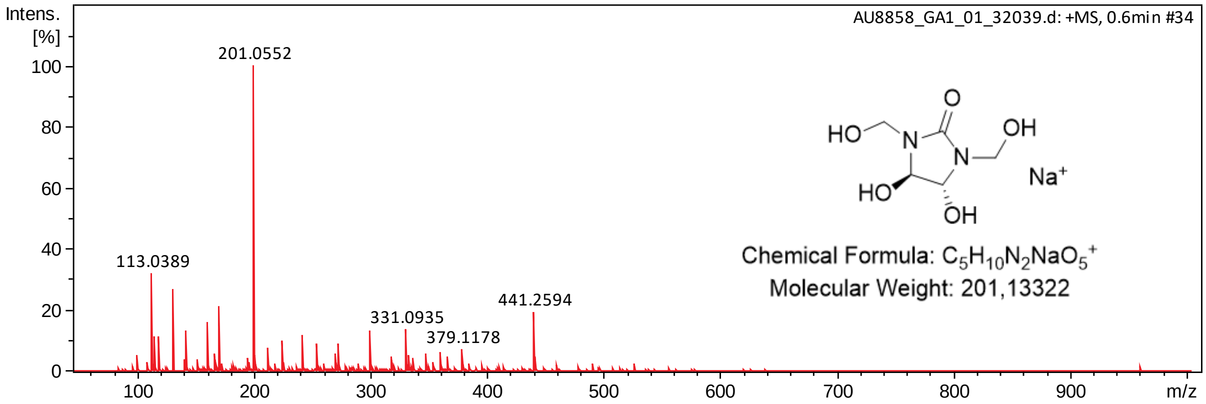

LCMS Data of compound **3** after reaction with HS⁻

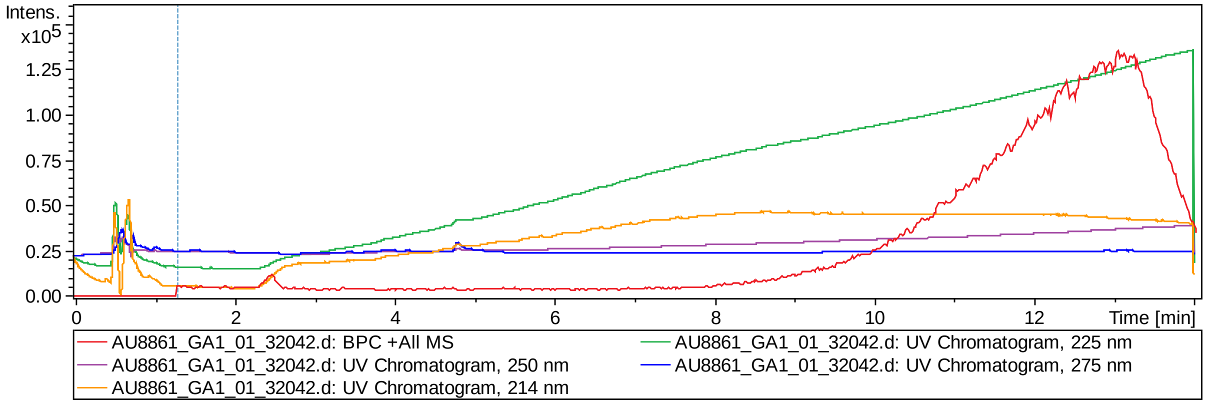

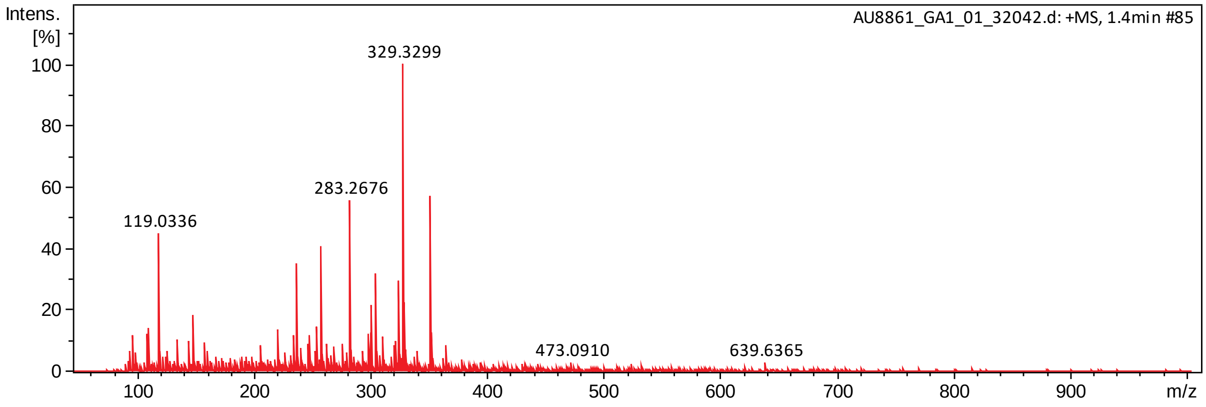

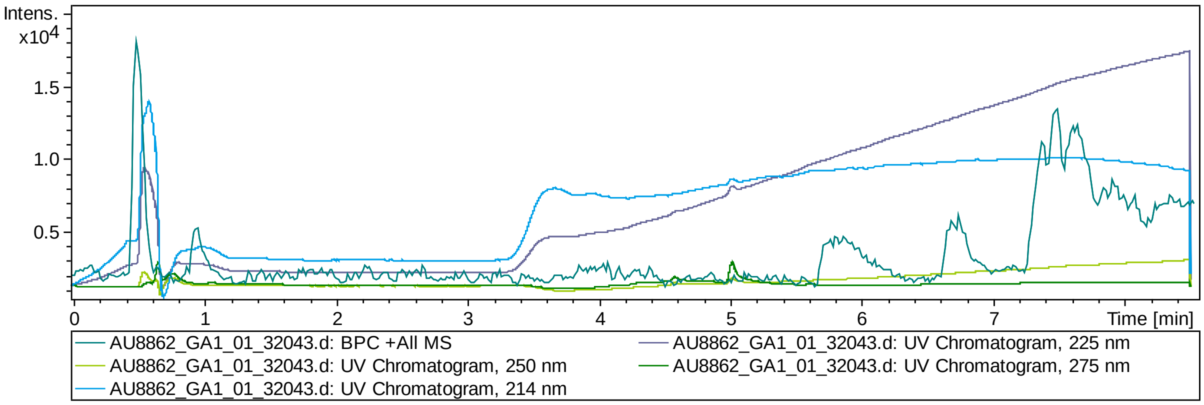

Intens.
x10^4
1.5
1.0
0.5
0
1
2
3
4
5
6
7
Time [min]
AU8862_GA1_01_32043.d: BPC +All MS
AU8862_GA1_01_32043.d: UV Chromatogram, 225 nm
AU8862_GA1_01_32043.d: UV Chromatogram, 250 nm
AU8862_GA1_01_32043.d: UV Chromatogram, 275 nm
AU8862_GA1_01_32043.d: UV Chromatogram, 214 nm

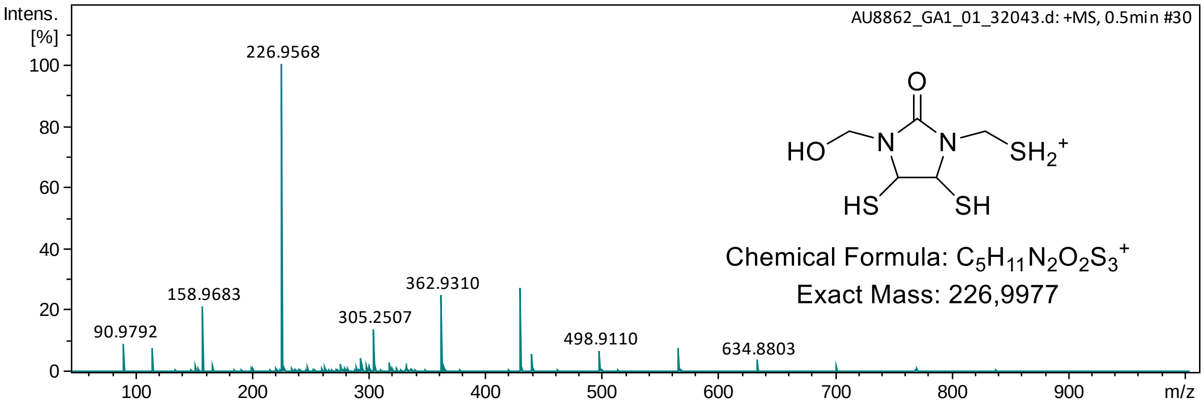

Intens.
[%]
AU8862_GA1_01_32043.d: +MS, 0.5min #30
226.9568
158.9683
90.9792
305.2507
362.9310
498.9110
634.8803
m/z
HO
N
O
SH2+
HS
SH
Chemical Formula: C5H11N2O2S3+
Exact Mass: 226,9977
